# Comprehensive sexuality education and adjacent prevention strategies for adolescent sexual violence: a scoping review of consent, empowerment and bystander action

**DOI:** 10.3389/fpubh.2026.1914237

**Published:** 2026-08-26

**Authors:** Fanni Hanifa, Nita Arisanti, Tetti Solehati, Rusnani Ab Latif, Meita Dhamayanti

**Affiliations:** 1 Faculty of Medicine, Universitas Padjadjaran, Bandung, Indonesia; 2 Midwifery Professional Education Study Program, Faculty of Vocational Studies, Universitas Indonesia Maju, South Jakarta, Indonesia; 3 Public Health Department, Faculty of Medicine, Universitas Padjadjaran, Bandung, Indonesia; 4 Department of Maternity Nursing, Faculty of Nursing, Universitas Padjadjaran, Bandung, Indonesia; 5 Department of Nursing, Faculty of Health Sciences, Universiti Teknologi MARA Cawangan Pulau Pinang, Head Batas, Pulau Pinang, Malaysia; 6 Department of Child Health, Faculty of Medicine, Universitas Padjadjaran, Bandung, Indonesia

**Keywords:** adolescent sexual violence, bystander intervention, comprehensive sexuality education, consent education, empowerment

## Abstract

**Introduction:**

Adolescent sexual violence is a persistent public-health, educational, and human-rights problem. Comprehensive Sexuality Education (CSE) is frequently proposed as a primary prevention approach, but evidence relevant to sexual violence is distributed across CSE, consent and respectful-relationships education, gender-transformative programming, empowerment, bystander intervention, and whole-school safeguarding. This scoping review mapped how CSE and explicitly related prevention strategies address adolescent sexual violence, which mechanisms and outcomes they emphasize and where evidence gaps remain.

**Methods:**

Following JBI guidance and PRISMA-ScR, 18 sources were charted and synthesized through a deductive–inductive thematic process.

**Results:**

The mapped sources comprised intervention evaluations, systematic and narrative reviews, qualitative and systems studies, program or adaptation papers, a protocol, and commentary. Four interconnected domains were identified: consent and relational ethics; empowerment, gender norms, and youth agency; bystander and peer mechanisms; and implementation and school-system conditions. The literature suggests that multicomponent approaches can improve knowledge, attitudes, communication, self-efficacy, and readiness to intervene; evidence for sustained reductions in perpetration or victimization is less consistent. Null or limited effects were reported for some behavioral outcomes, and communication diffusion did not necessarily produce behavioral diffusion. Most evidence came from high-income countries, with limited context-specific evidence from low- and middle-income countries.

**Discussion:**

CSE should therefore be understood as a defined curriculum-based, rights-oriented approach that may incorporate adjacent violence-prevention strategies without being equated with them. Future studies should use longitudinal behavioral outcomes, implementation and fidelity measures, intersectional analyses, and culturally grounded whole-school designs.

## 1 Introduction

Adolescence, defined here as the period from 10 to 19 years of age, is a formative phase in which rapid physical, cognitive, and psychosocial development intersects with emerging sexual scripts, peer norms, autonomy, and relationship practices (1, 2), Adolescent sexual violence is defined broadly as sexual acts, contact, or non-contact experiences that occur without freely given consent or when consent cannot be given; it includes sexual harassment, coercion, assault, forced sex and dating-related sexual violence (3). A public-health approach seeks to prevent the first occurrence by defining the problem, identifying risk and protective factors, developing and testing strategies, and supporting implementation at the population level (4).

Prevention during adolescence is justified by both developmental timing and empirical evidence. A meta-analysis of randomized psychosocial programs found modest reductions in adolescent sexual-violence perpetration and victimization, while emphasizing remaining risk-of-bias concerns and the need for stronger trials (5). Prevention therefore requires more than awareness: adolescents need practical capabilities to recognize coercion, communicate and respect boundaries, respond to peer pressure, identify unsafe situations, seek help, and support peers. These capabilities are consistent with evidence that interactive, skill-based and developmentally timed programs are more likely to affect behavior than information-only approaches (5–7).

UNESCO defines CSE as a curriculum-based process addressing the cognitive, emotional, physical, and social dimensions of sexuality. It is intended to develop knowledge, skills, attitudes, and values that support health, wellbeing, dignity, respectful relationships, rights, and protection from harm (8). CSE is therefore broader than biomedical instruction about pregnancy and infection, but it is not synonymous with every form of violence prevention. Consent education, respectful- or healthy-relationships education, gender-transformative education, bystander programs, dating-violence prevention, peer leadership, and youth–adult partnerships overlap with CSE in content or mechanism; they remain distinct program types unless they are integrated into a curriculum-based sexuality-education program. In this review, adjacent strategies were eligible only when they explicitly addressed sexual or dating violence in adolescent settings and contributed a mechanism directly relevant to CSE.

Comprehensive Sexuality Education (CSE) is a contested concept. It can be framed as a rights-based educational entitlement, a sexual and reproductive health intervention, a sexual-wellbeing approach, a primary-prevention strategy, or a combination of these purposes (8, 9). This review adopts a rights-based public-health framing while retaining conceptual boundaries: CSE is the curricular core, whereas adjacent programs are examined as complementary or potentially integrable prevention strategies. The term ‘multicomponent prevention approach’ is used instead of ‘prevention architecture’ to avoid implying that all included programs are forms of CSE.

Gender and power require explicit attention. Programs that address gender and power have shown stronger sexual and reproductive health outcomes than programs that omit these dimensions, while recent process evidence highlights facilitator skill, student participation, contextualization, and a supportive school and community environment (10, 11). Our Watch’s Change the Story framework identifies gender inequality, rigid gender roles, condoning of violence, male peer relations, victim-blaming, and institutional practices as interlocking drivers that must be changed across settings (12). This supports gender-transformative CSE that challenges entitlement and harmful masculinities, equips all students to recognize coercion, and links classroom learning with accountable school cultures.

Existing reviews establish important but different parts of this evidence base. Tibbels and Benbouriche (13) categorized 43 youth prevention studies by perpetration prevention, victimization-risk reduction, bystander intervention, and mixed approaches. Dos Santos et al. (14) focused on 15 school-based sexuality-education and sexual-violence-prevention studies published from 2019 to 2024. Schneider and Hirsch (15) proposed sequential K–12 CSE as a primary prevention strategy for sexual-violence perpetration but emphasized that CSE had not yet been evaluated using perpetration behavior as an outcome. The present review adds a different contribution: it explicitly distinguishes CSE from adjacent strategies, maps how consent, empowerment, gender transformation, bystander action, and school systems interact, and separates intervention evidence from qualitative, review, programmatic, and conceptual evidence so that claims are calibrated to evidence type.

The review questions were: (1) How has CSE, or an explicitly related adolescent prevention strategy, been used or proposed to prevent sexual violence? (2) Which consent, empowerment, gender-norm, bystander, peer, and institutional mechanisms are represented? (3) Which outcomes have been assessed, and what patterns of effect, non-effect, or contradiction are reported? and (4) What methodological, population, implementation, and geographic gaps remain? The purpose is evidence mapping and interpretive integration, not pooled effectiveness estimation or validation of a new causal framework.

## 2 Methods

### 2.1 Design, protocol and reporting

This scoping review was designed to map a broad and heterogeneous body of literature, clarify concepts, and identify evidence gaps rather than estimate a pooled intervention effect. The review followed the methodological framework of Arksey and O’Malley (16), refinements by Levac et al. (17), JBI guidance (18), and PRISMA-ScR reporting (19). No formal review protocol was developed, prospectively registered, or publicly published. Before screening, the review team defined the review questions, eligibility criteria, data-charting variables, and a synthesis approach. This unregistered protocol status is reported as a limitation.

### 2.2 Search strategy and information sources

The search used the Population-Concept-Context framework. The population was adolescents aged 10–19 years. Sources including participants aged 20–24 years or retrospective young-adult accounts were eligible only when their findings directly concerned adolescent or secondary-school prevention. The concept comprised CSE and adjacent educational strategies with an explicit component addressing the prevention of adolescent sexual or dating violence. Contexts included schools, community and youth-serving programs, and digital or blended interventions.

Searches were conducted from 28 September 2025 to 3 June 2026. Scopus was searched on 28 September 2025; Web of Science Core Collection on 22 October 2025; PubMed on 4 November 2025; ScienceDirect on 3 February 2026; Taylor & Francis Online on 7 April 2026; Sage Journals on 13 April 2026; and SpringerLink on 24 April 2026. PsycINFO and ERIC were searched during the final supplementary search completed on 3 June 2026. Backward and forward citation chaining was also completed on 3 June 2026. In consultation with the study team, an experienced individual conducted a comprehensive literature search. No librarian or information specialist was involved. No publication-year or country restriction was applied. Only English-language full-text scholarly sources were included. Grey literature, dissertations, theses, conference abstracts, unpublished reports, and sources without sufficient methodological, programmatic, or outcome information were excluded. Full database-specific strings are presented in Supplementary Table S1.

### 2.3 Eligibility criteria and conceptual boundary

Sources were included when they: (1) concerned adolescents aged 10–19 years, an adolescent-focused setting, or a youth sample from which adolescent-relevant evidence could be identified; (2) examined CSE, sexuality education, consent or respectful-relationships education, or an adjacent strategy such as dating-violence, bystander, empowerment, gender-transformative, male-youth, peer-leadership, or youth–adult partnership programming; (3) explicitly addressed prevention of sexual violence, sexual harassment, coercion, assault, unwanted sexual experiences, or dating-related sexual violence; and (4) provided sufficient information on population, program or mechanism, setting, or outcomes. School-, community-, and digital/online interventions were eligible. Setting was charted and considered during interpretation rather than treated as a basis for exclusion.

Sources were excluded if they focused only on adults with no adolescent relevance; addressed treatment after violence without a prevention component; concerned general reproductive health without an explicit violence-prevention link; lacked sufficient program or analytic detail; were grey literature or conference abstracts; or were unavailable in English. No study-design restriction was applied because empirical intervention studies, qualitative and mixed-methods studies, reviews, protocols, program descriptions, and conceptual contributions answer different mapping questions. These evidence types were not treated as equivalent: intervention evaluations informed outcome claims, whereas qualitative, systems, programmatic, and conceptual sources informed perceived needs, mechanisms, implementation conditions, or hypotheses.

### 2.4 Screening and selection

**Figure 1:**
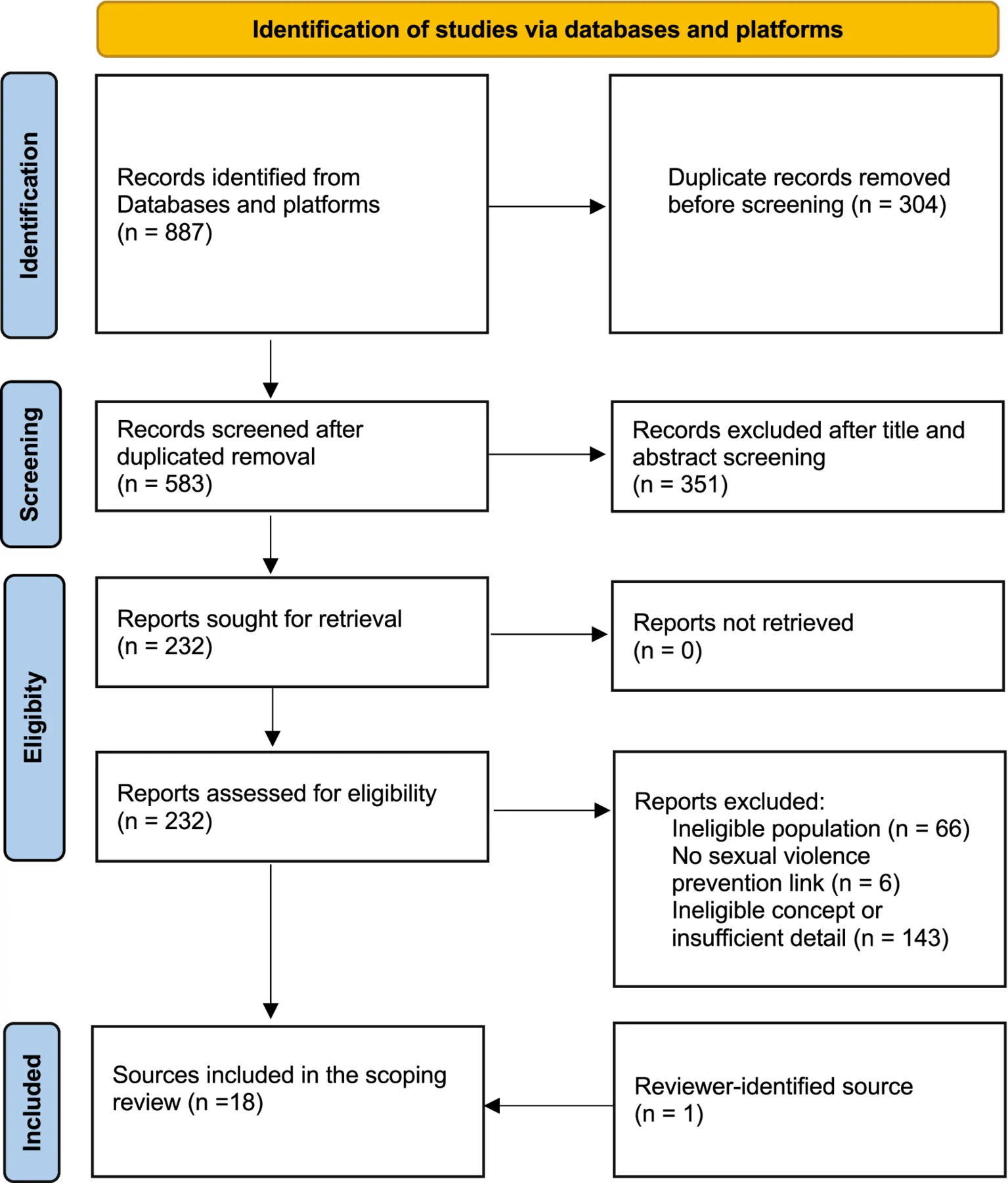
PRISMA-ScR flow diagram showing the reconciled identification, screening, eligibility, and inclusion of 18 sources (17 database-identified and one reviewer-identified).

Records were imported into Zotero and duplicates records were removed before screening. One member of the review team screened titles and abstracts and then assessed full texts. Screening was not performed independently by two reviewers; consequently, an inter-rater agreement statistic was not calculated. Uncertain decisions were discussed with the author team and resolved by consensus. The reconciled database-selection sequence was as follows: 887 records were identified; 304 duplicates were removed; 583 records were screened; 351 were excluded at title/abstract screening; all 232 reports sought for retrieval were obtained and assessed; 215 full-text reports were excluded (ineligible population, *n* = 66; no sexual-violence prevention link, *n* = 6; ineligible concept/source or insufficient detail, *n* = 143); and 17 sources were included. Following peer review, the reviewer-identified article by Schneider and Hirsch (15) was assessed against the same eligibility criteria and added to the evidence map, yielding a final total of 18 sources. The previously displayed value of 331 reports not retrieved reflected a flow-diagram labeling error rather than unavailable full texts (Figure 1).

### 2.5 Data charting and synthesis

A standardized charting form captured author and year, country and setting, evidence type and design, population and sample, program or exposure, CSE or consent content, empowerment and gender components, bystander or peer components, outcomes, findings, implementation issues, and limitations. One member of the review team charted all 17 sources; the author team reviewed the completed chart for completeness and consistency. Deductive categories were derived from the review questions and the conceptual distinctions set out in the Introduction, while inductive categories were generated from recurrent patterns in the charted evidence. The team compared, discussed, and refined the categories into four synthesis domains. Sources could contribute to more than one domain, and each claim was tagged by evidence type so that intervention evaluations, qualitative studies, reviews, protocols, program descriptions, and commentary were not treated as equivalent.

For analytic calibration, a mechanism was coded as evidence-derived only when an included source explicitly described, proposed, or evaluated it. Relationships among mechanisms across domains were developed during the authors’ synthesis and are labelled as interpretive integration rather than study-level findings.

### 2.6 Methodological appraisal

No formal risk-of-bias or critical-appraisal tool was applied, and sources were not excluded on methodological quality. This decision is consistent with the descriptive mapping purpose of the review and the optional status of critical appraisal in scoping reviews (18, 19). To support cautious interpretation, the charting form nevertheless recorded design, sample, follow-up, outcome type, implementation reporting, and source-specific limitations. Accordingly, the synthesis uses calibrated language and does not infer effectiveness from qualitative studies, commentaries, protocols, or program descriptions.

## 3 Results

Eighteen sources were charted. They included two primary intervention analyses (20, 21), one social-network evaluation (22), two qualitative or participatory systems studies (23, 24), four systematic or global reviews (14, 25–27), one narrative review of parenting programs (28), one practitioner-informed program review (29), two program or adaptation papers (30, 31), one trial protocol (32), one consent-focused narrative review (33), one CSE-focused conceptual review (15), one retrospective qualitative study of schooling (34), and one commentary (7). Most sources were from or synthesized evidence dominated by the United States, Australia, Scotland, and other high-income contexts. Kenya and Rwanda provided the clearest LMIC-specific empirical contexts.

The mechanism labels used below therefore summarize content represented in the included sources. The grouping of these mechanisms into four domains is the authors’ synthesis, and it should not be read as a causal model tested across the 18 sources (Table 1).

**Table 1:** Characteristics and analytical mapping of the 18 included sources.

Author / country-setting	Design / sample	Program or evidence focus	Prevention mechanisms	Outcomes or mapped factors	Main findings	Limitations / evidentiary role
Laha (33) India / narrative context	Narrative review; no participant sample	Consent education and healthy relationships	Consent, gendered power, coercion, entitlement	No empirical outcome	Clarifies consent as ongoing and contextual	Broad non-systematic review; cannot establish effectiveness
Schneider and Hirsch (15) USA / K–12 schools	Conceptual narrative review; no participant sample	NSES-guided K–12 CSE as primary prevention of sexual-violence perpetration	Early and sequential prevention; socioecological risk factors; gender and power; healthy relationships	No empirical outcome; proposed perpetration-prevention pathways	Argues that CSE may address multiple perpetration risk factors and calls for longitudinal evaluation	Conceptual source; CSE was not evaluated using perpetration behavior as an outcome; US-specific standards
Vrankovich et al. (34) Australia / school recollections	Qualitative; 20 adults aged 18–30 reflecting on school education	School sexuality education	Consent, pleasure, intimacy, ethics, safety	Perceived curricular adequacy	Participants described risk-focused teaching with limited practical preparation	Retrospective, small sample; contextual evidence, not intervention evaluation
Dos Santos et al. (14) International / schools	Systematic review; 15 studies (2019–2024)	School-based sexuality education and SV prevention	Consent, coercion, harassment, negotiation	Knowledge, attitudes, self-efficacy, intentions, behavior	Knowledge-related outcomes were more consistent than behavioral outcomes	Heterogeneity, short follow-up, weak intersectional and LGBTQIAPN+ inclusion
Orchowski et al. (21) USA / 26 high schools	Cluster trial; 2,685 Grade 10 students	Your Voice Your View workshops, teacher training, norms campaign	Victimization risk reduction, bystander skills, norms	Sexual aggression; unwanted intercourse	Small protective effect for unwanted intercourse; no significant sexual-aggression effect	Six-month follow-up; small effect; limited gender-diverse analysis
Blake et al. (23) Scotland / secondary schools	Participatory systems mapping; workshops *n* = 22; surveys *n* = 757; validation in 7 schools	School prevention, disclosure, reporting and response system	Knowledge, trust, communication, safeguarding, prioritization	25 mapped factors and 3 feedback loops	Shows how school systems condition reporting and response	Hypothesized systems map; not an effectiveness evaluation
Brodeur et al. (30) Canada / clinical-community adaptation	Intervention adaptation case study; no outcome sample	Dating-violence components added to a CSA intervention	Trauma-informed relationships and revictimization prevention	Adaptation process and practitioner validation	Identifies relational needs of adolescent girls with CSA histories	No comparative or behavioral outcome evaluation
Finnie et al. (26) High-income countries	Community Guide systematic review; 24 study arms	Youth intimate-partner and sexual-violence prevention	Relationship skills, norms, protective environments, bystander action	Perpetration, victimization, bystander outcomes	Eighteen of 24 study arms favored reduced perpetration; other outcomes were less consistent	Heterogeneous interventions and outcome definitions
Ellsberg et al. (25) Global / HIC and LMIC	Global best-practice review; no single participant sample	Adolescent intimate-partner and sexual-violence prevention	Gender transformation, autonomy, masculinities, structural factors	Program and policy evidence	Argues for gendered and structural approaches, particularly in LMICs	Diverse evidence types and measures limit direct comparison
Ybarra (7) USA / developmental prevention	Invited commentary; no participant sample	Early adolescent prevention programming	Developmental timing	Conceptual interpretation of meta-analytic evidence	Supports investment before norms and behaviors consolidate	Commentary; no new empirical data
Edwards et al. (31) USA / community agencies	Programmatic/theoretical article; no effectiveness sample	Girls’ Leadership Academy	Empowerment, leadership, wellbeing, relationships	Program theory and curriculum	Describes a practice-based empowerment model for girls	Behavioral effectiveness not established
Sarnquist et al. (32) Kenya / informal settlements	Cluster-RCT protocol; students aged 10–14	IMPower for girls plus Source of Strength for boys	Self-defense, positive masculinity, bystander action	Planned assault, self-efficacy, self-esteem, gender attitudes	Specifies coordinated girls’ and boys’ curricula	Protocol only; no results in this source; article carries an Expression of Concern
Verbeek et al. (27) International / male youth	Systematic review; 15 studies of 13 programs	Sexual and dating-violence prevention for males ≤25	Masculinity, attitudes, norms, behavioral control	Psychosexual and behavioral outcomes	Effects were more evident for longer-term behavior and short-term attitudes	Moderate-to-serious risk of bias; norms and control rarely measured
Kawonga et al. (24) Rwanda / community mentorship	Qualitative; 13 AGYW and 5 male community members	SRHR mentorship and community prevention	Empowerment, legal literacy, gender stereotypes	Perceived drivers and prevention strategies	Identified multilevel cultural, family, legal and substance-related drivers	Single district; small purposive sample; no intervention outcome
Marcus et al. (28) LMIC parenting programs	Narrative review; 17 violence/child-marriage initiatives from 42 programs	Parenting and caregiver interventions	Norm change, communication, male engagement	Violence-related norms and program reach	Participatory programs may shift norms when contextually embedded	Uneven evidence; limited scale and long-term maintenance
Edwards et al. (22) USA / middle and high schools	Social-network evaluation; 1,172 nonparticipant peers	Youth Voices in Prevention	Youth leadership and youth–adult partnership	Communication diffusion and violence-related behavior	Program discussion diffused through networks, but behavior did not	Diffusion alone was insufficient; dosage and reinforcement uncertain
Weisz and Black (29) USA / 52 programs	Practitioner-informed review of 52 programs	Peer education and youth leadership	Peer credibility and anti-violence norms	Practitioner-reported strengths and challenges	Peer leadership was viewed as engaging and empowering	Few formal outcome evaluations; dated US-focused evidence
Clear et al. (20) USA / Kentucky high schools	Secondary analysis of cluster RCT; 63,320 annual surveys	Green Dot bystander intervention	Bystander action and school norms	Self-reported pregnancy (indirect outcome)	Training receipt associated with lower pregnancy; randomized comparison was not significant	Indirect outcome; self-report; observational contrast may be confounded

### 3.1 Consent, relational ethics and CSE boundaries

Across the mapped literature, consent was treated as ongoing, voluntary, specific, reversible, and sensitive to power and context rather than as a one-time legal formula (33). Schneider and Hirsch (15) proposed sequential K–12 CSE as a population-level primary prevention strategy targeting perpetration risk factors through healthy-relationship content, social–emotional skills, attention to gender and power, and developmentally timed instruction. However, their conceptual review explicitly noted that CSE had not been evaluated using sexual-violence perpetration behavior as an outcome. Retrospective qualitative evidence indicated that young adults experienced school sexuality education as risk-focused and insufficiently practical for navigating consent, intimacy, pleasure, ethics, and safety (34). This finding identifies perceived curricular gaps but does not demonstrate intervention effectiveness. Similarly, participatory systems mapping in Scottish schools showed that knowledge of consent is only one part of prevention; disclosure and response were also shaped by trust, confidentiality, safeguarding, communication, and institutional prioritization (23).

Intervention and review evidence was more mixed. Your Voice Your View combined workshops, teacher training, and social-norm messaging; it produced a small protective effect for unwanted sexual intercourse at 6 months but no significant reduction in sexual aggression (21). Dos Santos et al. (14) reported that school-based interventions improved knowledge about consent and sexual health more consistently than behavior or violence outcomes. Finnie et al. (26) found more favorable patterns for perpetration than for victimization or sustained bystander outcomes across heterogeneous youth interventions. These findings support the inclusion of consent within CSE while cautioning against assuming that consent knowledge alone reduces violence.

Cross-study synthesis: qualitative, systems, and review evidence converged in treating consent as necessary, relational, and power-sensitive, but intervention evidence diverged on whether improved consent knowledge translated into less aggression or victimization. Collectively, the evidence supports consent as a core curricular mechanism within CSE, not as a sufficient stand-alone violence-prevention intervention.

### 3.2 Empowerment, gender transformation, and youth agency

Empowerment-oriented sources emphasize agency, self-efficacy, leadership, and relationship skills. The Girls’ Leadership Academy described a theoretically grounded community program integrating wellbeing, healthy relationships, leadership, and anti-violence attitudes, but it did not report an effectiveness evaluation (31). Brodeur et al. (30) documented a trauma-informed adaptation of dating-violence prevention for adolescent girls with child sexual abuse histories, its contribution concerns intervention design and acceptability rather than outcomes. The Nairobi protocol combined girls’ empowerment and self-defense with a boys’ curriculum addressing positive masculinity and bystander action, but the protocol itself provided no effectiveness results and therefore was not used to support effect claims (32).

Gender-transformative evidence pointed beyond individual self-protection. Reviews emphasized restricted autonomy, unequal relationship power, harmful masculinities, and peer or institutional norms (25, 27). The male-youth review found some behavioral and attitudinal effects but also moderate-to-serious risk of bias and limited measurement of social norms and perceived behavioral control (27). Qualitative evidence from Rwanda identified substance use, transactional relationships, inadequate family protection, gender stereotypes, limited legal literacy, and interference with justice processes; these data support culturally grounded hypotheses and program design, not causal claims about mentorship effectiveness (24).

Cross-study synthesis: sources converged in locating empowerment within agency, relational power, gender norms, and institutional context rather than individual self-protection alone. They diverged in evidentiary strength because several sources were protocols, program descriptions, or qualitative studies rather than outcome evaluations. Collectively, the evidence supports these mechanisms as theoretically and contextually plausible, while causal evidence for sustained violence reduction remains limited.

### 3.3 Bystander action, peer leadership, and social norms

Bystander and peer strategies redistributed responsibility from potential victims to peers and school communities. However, readiness and communication did not consistently translate into behavior. In Youth Voices in Prevention, discussion of the program diffused through peer networks, but behavioral outcomes did not diffuse in parallel (22). The practitioner-informed review of 52 peer programs described credibility, engagement, and leadership benefits, yet noted a scarcity of formal outcome evaluation (29). These sources suggest that peer visibility is a mechanism requiring adequate dosage, supervision, reinforcement, and institutional support rather than a stand-alone solution.

Green Dot provides an additional caution about outcome interpretation. Across 63,320 annual student surveys, reported training receipt was associated with lower pregnancy rates, but the randomized comparison was modest and not statistically significant; pregnancy was also an indirect outcome rather than a direct measure of sexual violence (20). Across broader reviews, perpetration outcomes were more consistently favorable than victimization and bystander outcomes, and longer follow-up sometimes showed attenuated bystander effects (26).

Cross-study synthesis: peer and bystander approaches converged on the value of credible messengers, shared responsibility, and visible norm change. Findings diverged at the behavioral level: communication and training exposure did not consistently produce direct or diffused reductions in violence-related outcomes. Collectively, the evidence supports peer and bystander components as reinforced elements of a multicomponent strategy, contingent on dosage, supervision, and institutional follow-through.

### 3.4 Evidence gaps and implementation conditions

The mapped literature was geographically and methodologically uneven. Evidence from high-income countries predominated, whereas Kenya and Rwanda supplied the clearest LMIC-specific empirical contexts, and Marcus et al. (28) synthesized parenting initiatives across LMICs. Cultural norms, legal literacy, poverty, family communication, gendered power, and institutional capacity differed substantially across settings, limiting global generalization. Digital or online interventions were eligible, but none of the 18 included sources evaluated a fully digital CSE intervention; this constitutes a specific evidence gap.

Implementation challenges included teacher and facilitator preparedness, confidence in discussing consent and gender, curriculum fidelity and adaptation, time and dosage, parental or community resistance, unclear safeguarding pathways, weak confidentiality, and inconsistent policy support. Few sources linked implementation measures to long-term perpetration or victimization outcomes. Outcome definitions also varied across harassment, coercion, unwanted intercourse, assault, dating violence, and indirect reproductive-health indicators. These differences constrain comparison and help explain why knowledge and attitude gains were more consistent than sustained behavioral effects.

Cross-study synthesis: across domains, the literature consistently identified implementation conditions as important, but few studies measured them alongside long-term perpetration or victimization. The evidence therefore supports whole-school capacity, safeguarding, fidelity, and cultural adaptation as enabling conditions; it does not yet establish which implementation package is sufficient across settings. Table 2 summarizes how each synthesis domain addresses the review questions and the type of evidence contributed.

**Table 2:** Alignment of synthesis domains with review questions.

Synthesis domain	Review question(s)	Evidence contribution
Consent and relational ethics	RQ1–RQ3	Defines CSE/adjacent content; maps consent-related outcomes and limits
Empowerment, gender, and agency	RQ1–RQ3	Maps agency, gender transformation, perpetration and victimization mechanisms
Bystander, peer, and norms	RQ1–RQ3	Maps peer mechanisms, diffusion, direct/indirect behavioral outcomes
Evidence gaps and implementation	RQ3–RQ4	Maps null or mixed findings, fidelity, setting, equity, geography, and outcome gaps

## 4 Discussion

The mapped literature suggests that CSE can contribute to adolescent sexual-violence prevention when its curriculum explicitly addresses consent, power, gender, communication, help-seeking, and ethical relationships and when it is reinforced by peer, family, and institutional conditions. This conclusion is intentionally narrower than treating all included programs as CSE or claiming that a single multicomponent model has been proven effective. CSE is the curriculum-based core; bystander, empowerment, male-youth, parenting, peer-leadership, and safeguarding strategies are adjacent approaches that may be integrated with or reinforce it.

The clearest pattern was the gap between proximal and behavioral outcomes. Knowledge, attitudes, perceived readiness, and communication were more consistently improved than perpetration or victimization. Some findings were explicitly null or limited: Your Voice Your View did not reduce sexual aggression, Youth Voices in Prevention generated communication without behavioral diffusion, and Green Dot’s randomized comparison for the indirect pregnancy outcome was not statistically significant (20–22). These contradictions are substantively important. They indicate that awareness and favorable norms are intermediate mechanisms whose translation into behavior depends on opportunity, reinforcement, dosage, and institutional response (35).

Recent evidence outside the 18-source evidence map provides additional context for digital delivery. A systematic review of adolescent sexual health during the COVID-19 pandemic found that online sexual and reproductive health interventions improved communication, knowledge, and attitudes and reduced acceptance of dating violence, while also identifying persistent inequities and gaps in service access (36). Because that review addressed adolescent sexual health broadly rather than sexual-violence prevention alone, it supports digital delivery as a complementary modality but does not constitute direct evidence that CSE reduces perpetration or victimization.

A gender-transformative orientation remains necessary but should not be reduced to messaging about individual attitudes. Change the Story emphasizes that violence is sustained through gendered norms, power imbalances, male peer relations, victim-blaming, and institutional practices (12). In practical terms, CSE should combine skills for all students with perpetration prevention, equitable masculinities, critical reflection on power, and visible institutional accountability. Empowerment and resistance skills may be protective, but they should never imply that girls or potential victims carry responsibility for preventing violence.

Whole-school implementation is therefore central. Teachers and facilitators need content knowledge, comfort, pedagogical skill, referral competence, and ongoing supervision. Schools also require confidential reporting pathways, safeguarding protocols, consistent disciplinary and support practices, student participation, and policy-level endorsement. Cultural adaptation must preserve core rights and prevention mechanisms while engaging families, community leaders, and local understandings of relationships, gender, and help-seeking. Adaptation without fidelity may dilute mechanisms; rigid fidelity without cultural responsiveness may undermine acceptability and reach. Implementation-effectiveness designs are needed to examine this balance.

Generalizability is limited by the concentration of evidence in the United States, Australia, Scotland, and other high-income settings. Rwanda and Kenya illustrate how poverty, legal literacy, transactional relationships, family protection, and justice-system processes can reshape both risk and feasible prevention (24, 32). LMIC-focused work should avoid importing high-income curricula without formative research, language adaptation, youth participation, assessment of teacher capacity, and alignment with local safeguarding and referral systems. More evidence is also needed for LGBTQIAPN+ adolescents, adolescents with disabilities, racialized and displaced youth, out-of-school adolescents, and those with prior trauma.

The review’s novelty lies in conceptual differentiation and evidence calibration. Unlike Dos Santos et al. (14), which reviewed recent school-based sexuality-education interventions, and Tibbels and Benbouriche (13), which classified a broader set of prevention programs by prevention target, this review maps points of connection and distinction between CSE and adjacent strategies and connects evidence-reported mechanisms to school-system conditions. It also identifies which claims arise from intervention evaluations versus reviews, qualitative studies, program descriptions, a protocol, or commentary. The four-domain map is an author-generated organizing interpretation of heterogeneous evidence, not a mechanism validated by the included studies or a tested causal framework.

This review has several limitations. No formal review protocol was developed or prospectively registered. Screening was conducted by one reviewer, although uncertain decisions were discussed with the author team. The English-language full-text restriction and exclusion of grey literature and non-English evidence may have introduced language and publication bias. The search strategy was developed without a librarian or information specialist. The 18 sources were heterogeneous in design, population, setting, and outcome and were dominated by high-income contexts. No formal risk-of-bias appraisal was conducted. Several included sources were non-intervention evidence, one was a protocol, and definitions of harassment, coercion, assault, dating violence, perpetration, and victimization varied. Digital interventions and long-term behavioral outcomes were scarce. Consequently, the review maps the field but cannot provide a pooled effect estimate or definitive claims of effectiveness.

## 5 Conclusion

The mapped evidence suggests that CSE can contribute to preventing adolescent sexual violence when it is explicitly rights-based, gender-transformative, skill-oriented, and connected to safe institutional systems. CSE should remain conceptually distinct from, while potentially incorporating or coordinating with, consent education, empowerment, bystander intervention, peer leadership, parenting, and whole-school safeguarding.

The strongest and most consistent findings concern knowledge, attitudes, communication, self-efficacy, and perceived readiness. Evidence for sustained reductions in perpetration or victimization is less consistent, and several mapped studies reported null, small, indirect, or non-diffusing behavioral effects. Future research should prioritize longitudinal and culturally grounded designs, validated and clearly differentiated violence outcomes, implementation and fidelity measures, intersectional subgroup analyses, LMIC leadership, and transparent reporting. Practice should pair curriculum with trained educators, youth participation, caregiver and community engagement, confidential reporting, survivor-responsive referral, and institutional accountability.

## Data Availability

The original contributions presented in the study are included in the article/supplementary material, further inquiries can be directed to the corresponding author/s.
