# Supplementary material for: Comprehensive sexuality education and adjacent prevention strategies for adolescent sexual violence: a scoping review of consent, empowerment and bystander action
Source: Front Public Health. 2026 Aug 26;14:1914237. doi: 10.3389/fpubh.2026.1914237 (PMC13553753; doi:10.3389/fpubh.2026.1914237)
Supplement: Supplementary file 1 [file Supplementary_File_1.pdf]

Supplementary Material

Supplementary Table S1. Database- and platform-specific search strategies

**Source**	**Search date**	**Search string**
Scopus	28/09/2025	TITLE-ABS-KEY("comprehensive sexuality education" OR "sexuality education" OR "sex education" OR "relationship* education" OR "consent education" OR "healthy relationship*") AND ("sexual violence" OR "sexual assault" OR "sexual coercion" OR "sexual harassment" OR "dating violence" OR "intimate partner violence") AND (adolescen* OR youth OR "young people" OR "high school*" OR "secondary school*") AND (prevent* OR "primary prevention" OR bystander* OR empowerment OR "gender transform*")
Web of Science Core Collection	22/10/2025	TS=("comprehensive sexuality education" OR "sexuality education" OR "sex education" OR "relationship* education" OR "consent education" OR "healthy relationship*") AND ("sexual violence" OR "sexual assault" OR "sexual coercion" OR "sexual harassment" OR "dating violence" OR "intimate partner violence") AND (adolescen* OR youth OR "young people" OR "high school*" OR "secondary school*") AND (prevent* OR "primary prevention" OR bystander* OR empowerment OR "gender transform*")
PubMed	04/11/2025	("comprehensive sexuality education" OR "sexuality education" OR "sex education" OR "relationship* education" OR "consent education" OR "healthy relationship*")[Title/Abstract] AND ("sexual violence" OR "sexual assault" OR "sexual coercion" OR "sexual harassment" OR "dating violence" OR "intimate partner violence") AND (adolescen* OR youth OR "young people" OR "high school*" OR "secondary school*") AND (prevent* OR "primary prevention" OR bystander* OR empowerment OR "gender transform*")
PsycINFO	03/06/2026	TI,AB(("comprehensive sexuality education" OR "sexuality education" OR "sex education" OR "relationship* education" OR "consent education" OR "healthy relationship*") AND ("sexual violence" OR "sexual assault" OR "sexual coercion" OR "sexual harassment" OR "dating violence" OR "intimate partner violence") AND (adolescen* OR youth OR "young people" OR "high school*" OR "secondary school*") AND (prevent* OR "primary

		prevention" OR bystander* OR empowerment OR "gender transform*"))
ERIC	03/06/2026	("comprehensive sexuality education" OR "sexuality education" OR "sex education" OR "relationship* education" OR "consent education" OR "healthy relationship*") AND ("sexual violence" OR "sexual assault" OR "sexual coercion" OR "sexual harassment" OR "dating violence" OR "intimate partner violence") AND (adolescen* OR youth OR "young people" OR "high school*" OR "secondary school*") AND (prevent* OR "primary prevention" OR bystander* OR empowerment OR "gender transform*")
ScienceDirect	03/02/2026	("comprehensive sexuality education" OR "sexuality education" OR "sex education" OR "relationship* education" OR "consent education" OR "healthy relationship*") AND ("sexual violence" OR "sexual assault" OR "sexual coercion" OR "sexual harassment" OR "dating violence" OR "intimate partner violence") AND (adolescen* OR youth OR "young people" OR "high school*" OR "secondary school*") AND (prevent* OR "primary prevention" OR bystander* OR empowerment OR "gender transform*")
Taylor & Francis Online	07/04/2026	("comprehensive sexuality education" OR "sexuality education" OR "sex education" OR "relationship* education" OR "consent education" OR "healthy relationship*") AND ("sexual violence" OR "sexual assault" OR "sexual coercion" OR "sexual harassment" OR "dating violence" OR "intimate partner violence") AND (adolescen* OR youth OR "young people" OR "high school*" OR "secondary school*") AND (prevent* OR "primary prevention" OR bystander* OR empowerment OR "gender transform*")
Sage Journals	13/04/2026	("comprehensive sexuality education" OR "sexuality education" OR "sex education" OR "relationship* education" OR "consent education" OR "healthy relationship*") AND ("sexual violence" OR "sexual assault" OR "sexual coercion" OR "sexual harassment" OR "dating violence" OR "intimate partner violence") AND (adolescen* OR youth OR "young people" OR "high school*" OR "secondary school*") AND (prevent* OR "primary prevention" OR bystander* OR empowerment OR "gender transform*")

SpringerLink	24/04/2026	("comprehensive sexuality education" OR "sexuality education" OR "sex education" OR "relationship* education" OR "consent education" OR "healthy relationship*") AND ("sexual violence" OR "sexual assault" OR "sexual coercion" OR "sexual harassment" OR "dating violence" OR "intimate partner violence") AND (adolescen* OR youth OR "young people" OR "high school*" OR "secondary school*") AND (prevent* OR "primary prevention" OR bystander* OR empowerment OR "gender transform*")
